# Alkaloid ligands enable function of homomeric human α10 nicotinic acetylcholine receptors

**DOI:** 10.3389/fphar.2022.981760

**Published:** 2022-09-16

**Authors:** Arik J. Hone, J. Michael McIntosh

**Affiliations:** ^1^ School of Biological Sciences, University of Utah, Salt Lake City, UT, United States; ^2^ MIRECC, George E. Whalen Veterans Affairs Medical Center, Salt Lake City, UT, United States; ^3^ Department of Psychiatry, University of Utah, Salt Lake City, UT, United States; ^4^ George E. Whalen Veterans Affairs Medical Center, Salt Lake City, UT, United States

**Keywords:** α10 nicotinic acetylcholine receptors, α-conotoxin RgIA, strychnous nux vomica, neuropathic pain, chronic inflammation

## Abstract

In the nervous system, nicotinic acetylcholine receptors (nAChRs) rapidly transduce a chemical signal into one that is electrical via ligand-gated ion flux through the central channel of the receptor. However, some nAChR subunits are expressed by non-excitable cells where signal transduction apparently occurs through non-ionic mechanisms. One such nAChR subunit, α10, is present in a discreet subset of immune cells and has been implicated in pathologies including cancer, neuropathic pain, and chronic inflammation. Longstanding convention holds that human α10 subunits require co-assembly with α9 subunits for function. Here we assessed whether cholinergic ligands can enable or uncover ionic functions from homomeric α10 nAChRs. *Xenopus laevis* oocytes expressing human α10 subunits were exposed to a panel of ligands and examined for receptor activation using voltage-clamp electrophysiology. Functional expression of human α10 nAChRs was achieved by exposing the oocytes to the alkaloids strychnine, brucine, or methyllycaconitine. Furthermore, acute exposure to the alkaloid ligands significantly enhanced ionic responses. Acetylcholine-gated currents mediated by α10 nAChRs were potently inhibited by the snake toxins α-bungarotoxin and α-cobratoxin but not by α-conotoxins that target α9 and α9α10 nAChRs. Our findings indicate that human α10 homomers are expressed in oocytes and exposure to certain ligands can enable ionic functions. To our knowledge, this is the first demonstration that human α10 subunits can assemble as functional homomeric nAChRs. These findings have potential implications for receptor regulatory-mechanisms and will enable structural, functional, and further pharmacological characterization of human α10 nAChRs.

## Introduction

Nicotinic acetylcholine receptors (nAChRs) are composed of five individual subunits and form an ion channel that can be gated by acetylcholine (ACh) and related ligands (Dani 2015). Most heteromeric nAChRs, such as the α4β2 subtype, are composed of α and β subunits, whereas homomeric subtypes including α7 and α9 nAChRs are composed of a single gene product. Since the discovery of the *CHRNA10* gene approximately 20 years ago, attempts to heterologously express mammalian α10 subunits as functional homomeric receptors have consistently failed in both mammalian and non-mammalian expression systems (Elgoyhen, Vetter et al., 2001; Lustig, Peng et al., 2001; Sgard, Charpantier et al., 2002). By contrast, chick and frog α10 subunits do form functional homopentamers (Lipovsek, Fierro et al., 2014; Marcovich, Moglie et al., 2020). Furthermore, mammalian α9 subunits also form functional receptors in oocytes and mammalians cell lines although expression levels are generally low (Elgoyhen, Johnson et al., 1994; Filchakova and McIntosh 2013). These observations led to the conclusion that mammalian α10 subunits do not form functional receptors in the absence of α9 subunits but form α9α10 heteromers when the two subunits are expressed together.

The distribution of α10 subunits in mammalian organisms has a very restricted expression pattern and is limited to tissues outside the central nervous system (Elgoyhen, Vetter et al., 2001; Morley, Whiteaker et al., 2018). In humans, the presence of mRNA for *CHRNA10* or immunohistochemical evidence of α10 subunits has been demonstrated in pituitary and cochlear tissue (Sgard, Charpantier et al., 2002), immune cells, including monocytes (Richter, Mathes et al., 2016) and lymphocytes (Lustig, Peng et al., 2001; Peng, Ferris et al., 2004), and certain epithelial tissues such as urothelium (Bschleipfer, Schukowski et al., 2007) and skin (Kurzen, Berger et al., 2004). *CHRNA10* mRNA has also been detected in human breast and lung cancer-derived cell lines (Lee, Huang et al., 2010; Mucchietto, Fasoli et al., 2018). In rodents, mRNA for *CHRNA10* has been detected in pituitary (Hone, Rueda-Ruzafa et al., 2020), hair cells of the cochlea (Elgoyhen, Vetter et al., 2001), dorsal root ganglion neurons (Hone, Meyer et al., 2012), and several immune cell types (Kawashima, Yoshikawa et al., 2007; Mikulski, Hartmann et al., 2010).

The functional role of α10 subunits outside the auditory system is mostly unknown, but studies using subtype-selective ligands have implicated α10-containing nAChRs in a number of pathophysiological processes. Several α9α10-targeting ligands have been shown to inhibit the release of the proinflammatory cytokine interleukin-1β from human U937 monocytes (Richter, Mathes et al., 2016; Zakrzewicz, Richter et al., 2017). In rodent models of nerve injury (Vincler, Wittenauer et al., 2006), inflammatory bowel disease (AlSharari, Toma et al., 2020), and chemotherapeutic-induced neuropathy (Pacini, Micheli et al., 2016; Gajewiak, Christensen et al., 2021) administration of antagonist ligands of α9α10 nAChRs has been shown to reduce signs and symptoms of disease. α9α10 antagonists have also been shown to accelerate functional recovery of damaged nerves in models of neuropathic pain (Satkunanathan, Livett et al., 2005). Thus, development of a non-opioid based analgesic that targets α9α10 nAChRs is an active area of research.

Mammalian α10 subunits have long been thought to require α9 subunits for functional expression, and therefore ligand-receptor interactions have usually been examined in the context of α9α10 heteromers (Perez, Cassels et al., 2009; Azam and McIntosh 2012; Yu, Kompella et al., 2013; Indurthi, Pera et al., 2014; Zouridakis, Papakyriakou et al., 2019). Consequently, information concerning the interaction of ligands with human α10 subunits is significantly limited. Plant alkaloids including strychnine (STR) and methyllycaconitine (MLA) are known ligands of nAChRs containing α9 or α10 subunits, and MLA has been shown to promote functional expression of human α9α10 heteromers in *Xenopus* oocytes and human embryonic kidney cells (Gu, Knowland et al., 2020). Strychnine and the related compound brucine (BRU) are the principal alkaloids found in the seeds of *Strychnos nux-vomica*, the strychnine tree (Guo, Wang et al., 2018; Lu, Huang et al., 2020). Interestingly, the seeds of this tree are widely consumed as alternative medicine in India and southeast Asia where the tree is endemic. Internet sales of nux-vomica seeds and extracts known as Kuchla are now promoting wider use for indications that include pain, gastrointestinal disorders, and erectile dysfunction (Ades, Alteri et al., 2009; Akbar, Khan et al., 2010; Lu, Huang et al., 2020). Here we demonstrate that, when exposed to *strychnos* alkaloids or the larkspur alkaloid MLA, human α10 subunits form functional nAChRs in oocytes. The information presented in this report will facilitate the study of a nAChR subunit implicated in a number of human disease states and will allow, for the first time, structural, pharmacological and functional characterization of homomeric human α10 nAChRs.

## Methods

### Peptide synthesis

α-Conopeptide synthesis was performed using Fmoc solid-phase synthesis techniques and described in detail elsewhere (Hone, Fisher et al., 2019). The masses of the peptides were verified by matrix-assisted laser desorption/ionization time-of-flight mass spectrometry. Correct folding of the peptides and purity were determined by reverse-phase high-performance liquid chromatography and purity was ≥95% for all.

### Oocyte electrophysiology

Protocols (No. 17-07020) for obtaining oocytes from *Xenopus laevis* frogs were approved by the University of Utah’s Institutional Animal Care and Use Committee. Frogs were purchased from Xenopus1 (Dexter, MI, United States) and maintained by university personnel in an AAALAC accredited facility. Oocytes were obtained from frogs that were anesthetized with 0.4% wt/vol Tricaine-S (Thermo Fisher Scientific, Waltham, MA, United States) and sacrificed after removal of the ovarian lobes. Methods for preparation of cRNA constructs for expression of nAChRs in oocytes have been previously described (Hone, Talley et al., 2018). Clones for human α9 and α10 subunits were obtained from L.R. Lustig (University of California San Francisco, San Francisco, CA, United States) and were subsequently subcloned into a pSGEM vector that contained an alfalfa mosaic virus sequence (Filchakova and McIntosh 2013). Stage IV-V oocytes were generally injected with 50 ng of cRNAs encoding human nAChR subunits and subjected to two-electrode voltage-clamp electrophysiology 3 to 5 days after injection. However, injection of 2 ng of α10 subunit cRNA yielded currents that were −740 ± 245 nA (n = 13) indicating that small amounts of cRNA are sufficient to induce robust expression of α10 nAChRs. The effects of ACh, choline, nicotine, α-Bgtx, MLA, STR, and BRU on the functional expression of α10 nAChRs were assessed 3 days after injection of 50 ng of cRNA. For these experiments, the oocytes were removed from the incubation solution, placed in the recording chamber and, after voltage-clamping the membrane, were stimulated with 1 mM ACh while continuously perfused with frog saline only. The terms incubation and treated are used interchangeably throughout the manuscript to indicate the presence of ligand in the saline solution used to maintain the oocytes while in the incubator.

For the assessment of ligand activity, the oocyte membranes were clamped at a holding potential of −70 mV and continuously perfused with saline (control solution). The oocytes were pulsed with ACh (1 mM) for 2 s, once per minute, until a stable baseline-response was observed, then the control solution was switched to one containing the ligand of interest and the ACh responses monitored for changes in amplitude. The ACh responses in the presence of the ligand were normalized to the average of three responses in control solution. Ligands were applied in this manner for concentrations up to 1 µM. For concentrations >1 μM, ligands were applied in a static bath for 5 min and the current amplitudes were normalized to the ACh response after a 5 min bath application of control solution. For the concentration-response experiments using STR, BRU, or MLA, the ligands were perfusion applied at all concentrations. The estimated IC_50_ value for inhibition of ACh-evoked currents by STR and α-Bgtx were obtained by non-linear regression using a four-parameter logistic equation Y=Bottom + (Top-Bottom)/(1 + 10^((LogEC_50_-X)*HillSlope)). The estimated EC_50_ value for activation of α10 nAChRs by ACh was obtained according to the following procedures: the oocytes were stimulated with 2 s pulses of ACh (1 mM) every 60 s, and after a steady-state baseline was observed, the oocytes were then stimulated with ascending concentrations of ACh. The estimated maximal response for activation by ACh in each cell was determined using a four-parameter logistic equation and the responses to all concentrations of ACh, choline, and nicotine were then normalized to this value and shown as a percent response. The estimated plateau value for experiments examining the decay of ACh-evoked currents was determined using a one-phase exponential equation. For experiments that assessed the permeability of α10 channels to Ca^2+^ and the contribution of endogenous calcium-activated chloride channels to the observed responses to ACh, frog saline was used where CaCl_2_ was replaced with an equimolar concentration of BaCl_2_. For experiments examining the desensitization kinetics of α10-containing nAChRs, sibling oocytes were injected with 25 ng of cRNA for α10 subunits or cRNA for α9 and α10 subunits (25 ng each). Actinomycin-D (1 µM) was also used in some experiments to prevent RNA transcription and inhibit the expression of endogenous chloride channels. The oocytes were incubated in actinomycin-D for 36 h prior to assessment with TEVC electrophysiology.

### Statistical analysis

All statistical analyses were performed using Prism 9 (RRID: SCR_002798) (GraphPad Software, San Diego, CA, United States). The error bars for all experiments indicate the SD of the data and are provided to assess variance of the data. The data were analyzed for normality using a Shapiro-Wilk test. Significant differences in the ACh responses after incubation with saline or saline containing nAChR ligands were determined using a Kruskal–Wallis test with a post hoc Dunn’s multiple comparisons test. A paired student’s t-test was used to determine differences in current amplitudes obtained from α10 nAChR expressing oocytes in the presence of Ca^2+^ compared to Ba^2+^. An unpaired student’s t-test was used to determine differences in α10 nAChR current amplitudes obtained in the presence 10 nM compared to 1 μM MLA, 100 nM BRU compared to 10 μM BRU, or 10 nM STR compared to 1 μM STR. A paired student’s t-test was also used to determine differences in current amplitudes for α9 nAChR expressing oocytes incubated in saline and reassessed 24 h later after incubation in saline containing STR. To evaluate the inhibitory effects of antagonist ligands on α10 nAChR, a one-sample *t*-test was used and the values compared to a hypothetical response mean of 100% except in the case of Vc1.1 where a non-parametric Wilcoxon signed-rank test was used. Results were considered significant if *p* < 0.05*, *p* < 0.01**, *p* < 0.001***, or *p* < 0.0001****.

### Materials

Acetylcholine chloride and methyllycaconitine citrate were obtained from Tocris (Minneapolis, MN, United States). Strychnine hydrochloride, dimethoxystrychnine sulfate hydrate (brucine), atropine sulfate monohydrate (-)-nicotine hydrogen tartrate, sodium chloride, potassium chloride, calcium chloride dihydrate, magnesium chloride hexahydrate, 4-(2-hydroxyethyl)-1-piperazineethanesulfonic acid (HEPES), actinomycin-D, α-Bgtx from *Bungurus multicinctus*, α-cobratoxin from *Naja kaouthia,* were obtained from Sigma-Aldrich (St. Louis, MO, United States).

## Results

### Strychnine, BRU, and MLA enable ionic functions of human α10 nAChRs

**FIGURE 1:**
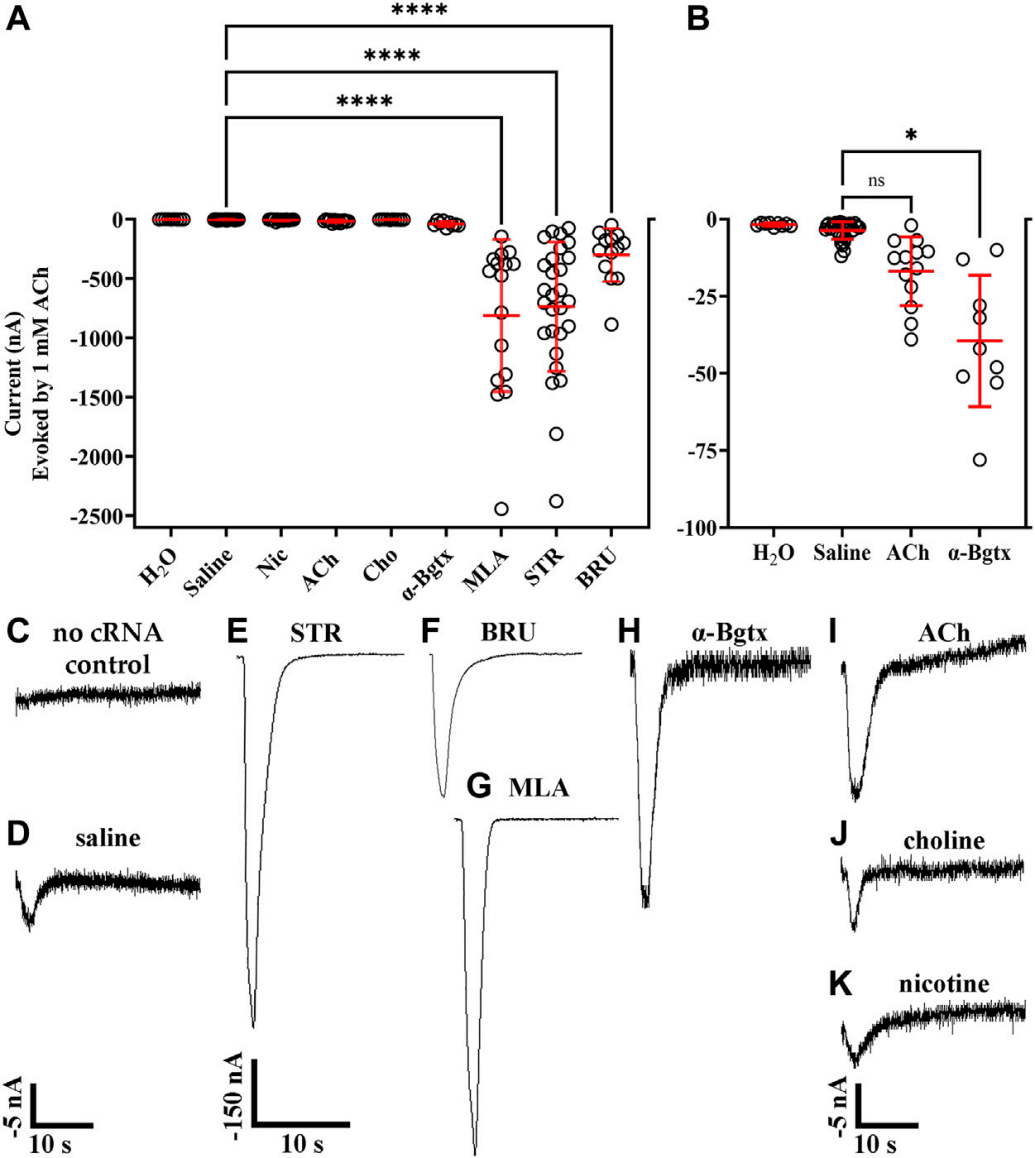
Ligand-binding promotes function of human α10 nAChRs expressed in *X. laevis* oocytes. Oocytes were injected with cRNA encoding human α10 subunits and treated for 3 days with frog saline or saline containing the indicated compounds then assessed by voltage-clamp electrophysiology for functional responses to acetylcholine (ACh; 1 mM). **(A)** Scatter plots of the data obtained under the indicated treatment conditions. **(B)** expanded data for oocytes injected with water, untreated (saline incubation only), and treated with saline containing ACh (5 mM) or α-Bgtx (1 μM). The noise amplitude recorded from oocytes injected with water was −1.7 ± 0.5 nA (n = 9). Oocytes injected with water containing cRNA for α10 subunits responded to ACh with current amplitudes that were −3.6 ± 2.8 nA (n = 32). Oocytes treated with STR (20 μM), BRU (20 μM) or MLA (20 μM) responded to ACh with current amplitudes that were significantly larger than those from controls; −735 ± 545 nA with a range of −76 to −2,378 nA (n = 28) for STR, −302 ± 225 nA with a range of −50 to −888 nA (n = 13) for BRU, and −813 ± 641 nA with a range of −146 to −2,443 nA (n = 16) for MLA. Oocytes treated with α-Bgtx, ACh, Cho, or Nic were −39 ± 21 nA (n = 9), −17 ± 11 nA (n = 13), −3.7 ± 1.5 nA (n = 10), and −6.0 ± 6.2 nA (n = 15), respectively. Currents from oocytes treated with α-Bgtx were slightly larger than saline controls, but there were no significant differences in current amplitudes recorded from oocytes treated with ACh, Cho or Nic compared to saline controls. **(D)** Example of a current trace from a control oocyte injected with water and stimulated with ACh. **(D–K)** Current traces from oocytes treated with the indicated ligand. The −5 nA scale bar applies to traces in **(C,D)**, and **(H–K)** and the −150 nA scale bar applies to **(E–G)**. The “±” and error bars indicate. The “n” indicates the number of oocytes obtained from 15 donors.

Certain nAChR ligands, most notably the tobacco alkaloid nicotine, have been shown to promote functional receptor expression in different cell types (Srinivasan, Pantoja et al., 2011; Henderson and Lester 2015). To determine if expression of human α10 nAChRs could be facilitated by exposure to alkaloids or other nicotinic ligands, we expressed α10 subunits in oocytes and exposed them to STR (20 μM), BRU (20 μM), acetylcholine (ACh; 5 mM), choline (5 mM), nicotine (20 μM), α-bungarotoxin (α-Bgtx; 1 μM), or MLA (20 μM) for 3 days. The oocytes were then stimulated with ACh (1 mM) under voltage-clamp conditions and assessed for functional responses. Oocytes injected with water and incubated in saline did not respond to ACh (Figures 1A–C). Very small or no ACh-evoked currents were observed in oocytes injected with cRNA for α10 subunits (−3.6 ± 2.8 nA; n = 32) (Figures 1A,B,D), but relatively large α10 nAChR currents were observed after incubating the oocytes in STR, BRU, or MLA (Figures 1A,E–G). Oocytes incubated with STR responded to ACh with large amplitude currents (−738 ± 545 nA; n = 28), but control oocytes injected with water and treated with STR did not respond to ACh (−1.6 ± 0.7 nA; n = 7). Current amplitudes recorded from oocytes incubated in BRU were −302 ± 225 nA (n = 13), and those from oocytes incubated in MLA were −813 ± 641 nA (n = 16). Small gains in current amplitudes were observed when the oocytes were incubated with ACh (−17 ± 11 nA; n = 13) or α-Bgtx (−31 ± 21 nA; n = 9), but only currents from those treated with α-Bgtx were significantly different than saline controls (Figures 1A,B,H,I). Currents recorded from oocytes incubated with choline (−7.2 ± 4.2 nA; n = 10) or nicotine (−6.0 ± 6.2 nA; n = 15) were not statistically different in amplitude than those from controls (Figures 1A,B,J,K).

**FIGURE 2:**
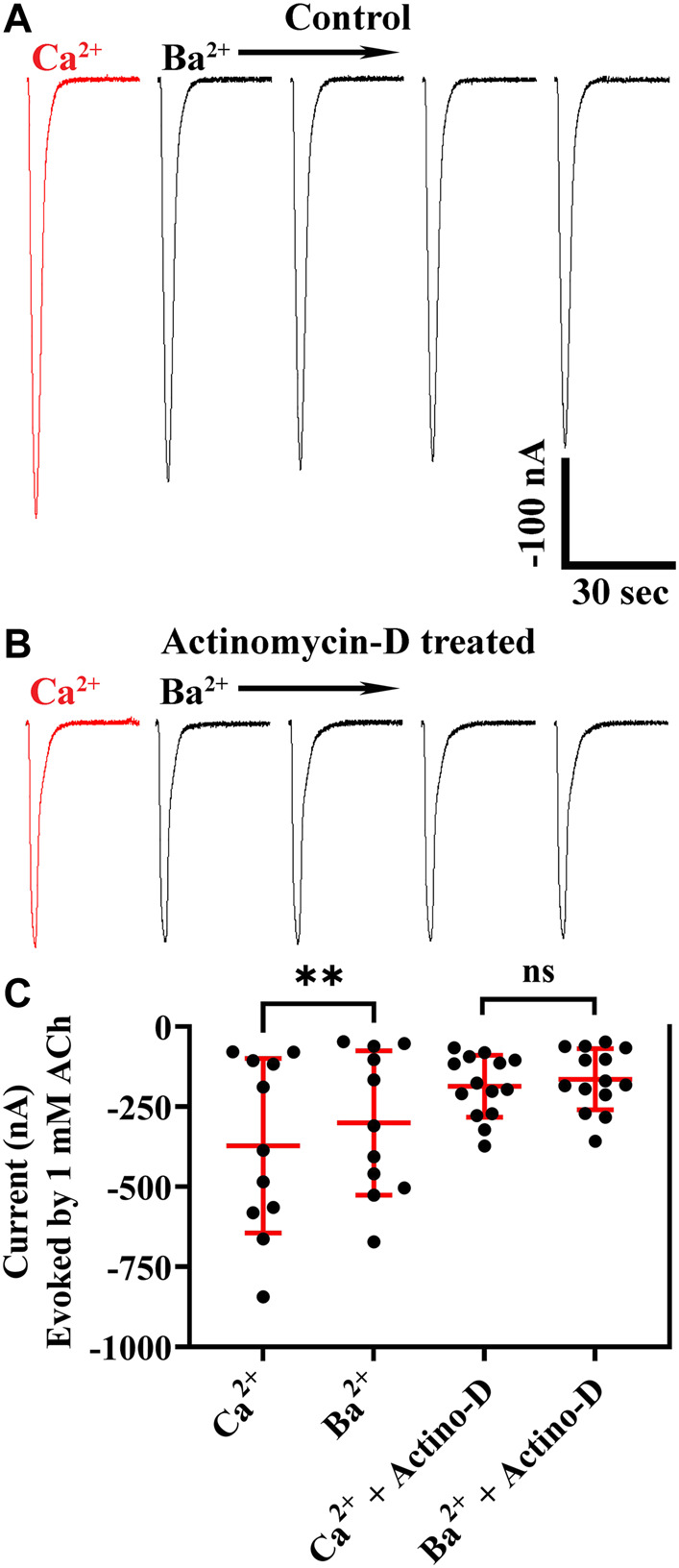
Human α10 nAChR currents are smaller in the presence of Ba^2+^ compared to those in Ca^2+^. Oocytes were injected with cRNA encoding human α10 subunits and treated for 36 h with frog saline containing STR (20 μM) and assessed by voltage-clamp electrophysiology for functional responses to acetylcholine (ACh; 1 mM) in the presence of calcium or barium. **(A)** Current traces from an oocyte perfused with saline containing calcium (1.8 mM) then with saline where calcium was replaced with equimolar barium. The responses in the presence of barium were smaller than those recorded in the presence of calcium (−373 ± 273 nA vs. −301 ± 225 nA, respectively; n = 11). The experiment was repeated with sibling oocytes that had been incubated with 1 µM actinomycin-D (Actino-D) to inhibit RNA transcription. **(B)** Current traces from an oocyte treated with Actino-D and perfused with saline containing calcium (1.8 mM) then with saline where calcium was replaced with equimolar barium. **(C)** No differences in response amplitudes in the presence of calcium compared those recorded in the presence of barium (−186 ± 96 nA vs. −164 ± 96 nA, respectively; n = 14) were found in oocytes treated with Actino-D. The current traces in **(A)** and **(B)** are shown with the 30 s the interpulse intervals reduced to 5 s for brevity and the scale bar in **(A)** also applies to **(B)**. The “±” and error bars indicate SD. The “n” indicates the number of oocytes obtained from two donors.

*Xenopus* oocytes express endogenous calcium-activated chloride channels, and Ca^2+^ entering the cell through calcium-permeable channels can contribute to currents in response to exogenously applied ligands (Barish 1983; Miledi and Parker 1984). To determine if these chloride channels contributed to the observed ACh-evoked currents, we conducted experiments by replacing external calcium (1.8 mM) with equimolar barium, an ion that does not activate chloride channels. Currents recorded in barium were smaller than those recorded in calcium (Figures 2A–C) indicating that activation of the ion channels formed from α10 subunits have less of an effect on *Xenopus* calcium-activated chloride channels compared to activation of mammalian α9-containing subtypes (Lipovsek, Fierro et al., 2014; Marcovich, Moglie et al., 2020). To further assess the potential contribution of chloride-channel activation to the ACh-evoked current amplitudes, we incubated oocytes exogenously expressing α10 nAChR subunits in actinomycin-D to inhibit RNA transcription and prevent expression of endogenous receptors and ion channels. The current amplitudes from oocytes treated with actinomycin-D and perfused with saline containing calcium were no different than those from sibling oocytes not exposed to actinomycin-D and perfused with barium saline (Figures 2B,C). Together, these results indicate that ACh evokes current through exogenously expressed α10 homomers both in the presence and in the absence of endogenous calcium-activated chloride channels.

### Strychnine, BRU, and MLA enhance functionality of α10 nAChRs but act as antagonists at high concentrations

**FIGURE 3:**
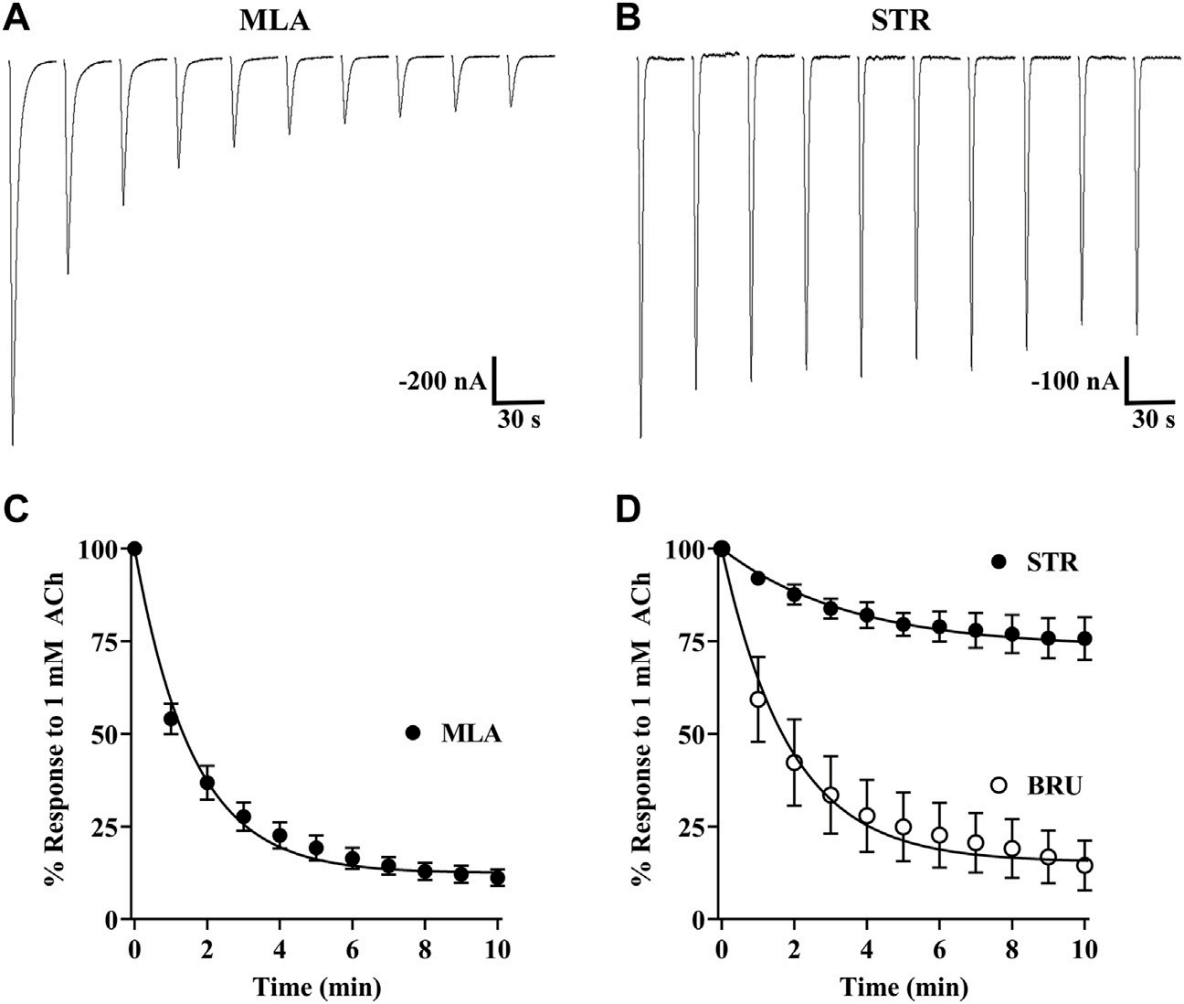
Strychnine, BRU, and MLA produce differential effects on α10 nAChR functionality. *X. laevis* oocytes were injected with cRNA encoding human α10 subunits and treated for 3 days in frog saline containing MLA (20 μM), STR (20 μM), or BRU (20 μM) and assessed by voltage-clamp electrophysiology. **(A)** Current traces from an oocyte treated with MLA and stimulated with acetylcholine (ACh; 1 mM) immediately after being placed in the recording chamber. **(B)** Current traces from an oocyte treated with STR and stimulated with ACh immediately after being placed in the recording chamber. **(C)** The calculated plateau values indicated that currents from oocytes treated with MLA would decay to 12 (11–13) % (n = 8) of initial current amplitudes. **(D)** Similarly, currents from oocytes treated with BRU would decay to 15 (13–17) % (n = 8) of initial values. By contrast, oocytes treated with STR decayed to only 74 (72–76) % (n = 13) of initial values. Representative current traces for MLA and STR are shown with the 30 s interpulse intervals reduced to 5 s for brevity. The error bars in **(C)** and **(D)** indicate SD and values in parenthesis indicate 95% CI. The “n” indicates the number of oocytes obtained from five donors.

**FIGURE 4:**
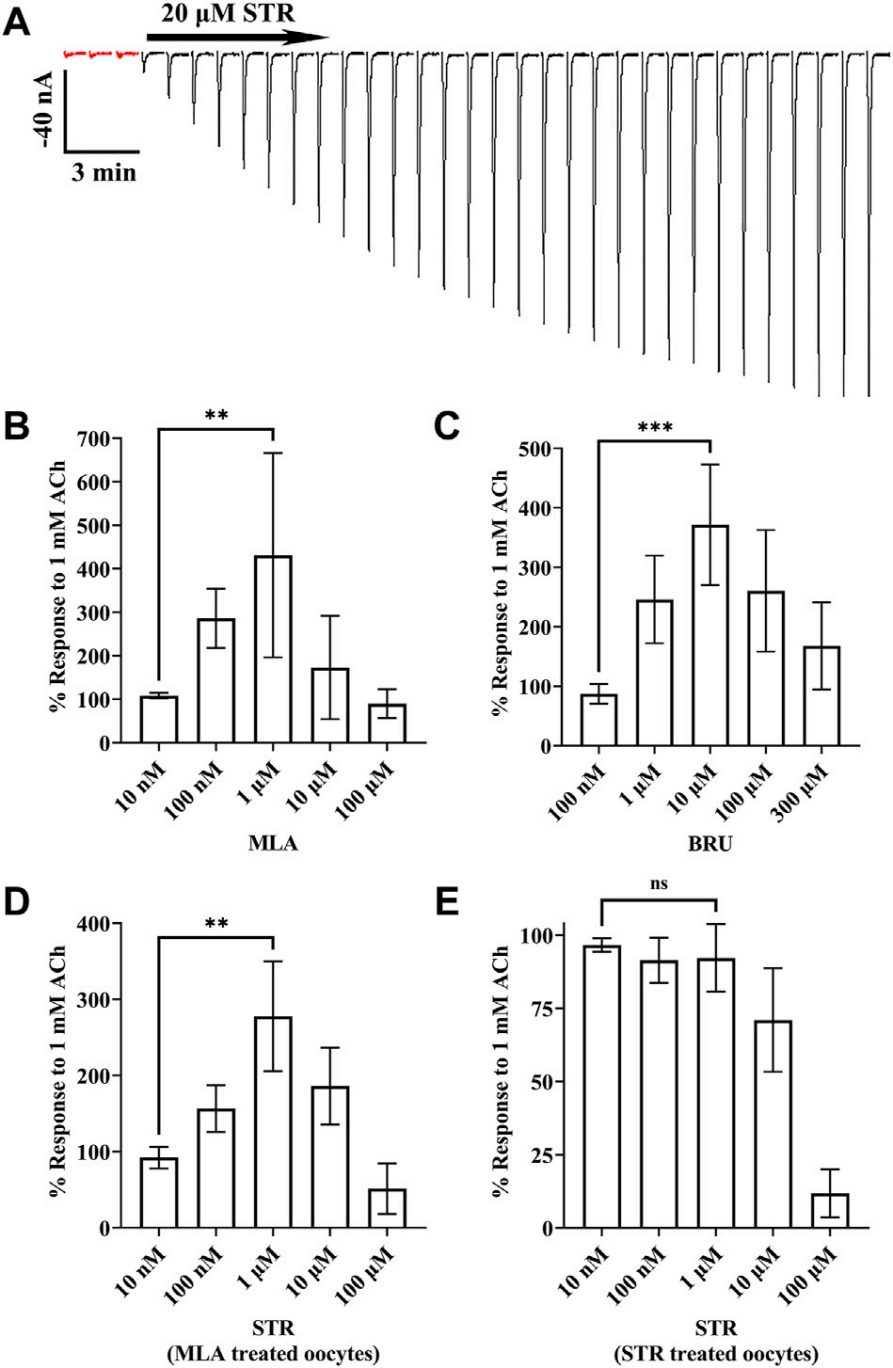
Currents evoked by ACh are enhanced by acute exposure to STR, BRU, or MLA. *X. laevis* oocytes were injected with cRNA encoding human α10 subunits and incubated for 3–4 days with frog saline then assessed by voltage-clamp electrophysiology. **(A)** Oocytes incubated in saline were stimulated with acetylcholine (ACh; 1 mM) and acutely perfused with STR (20 μM) for 30 min. The responses to ACh before and after perfusion with STR were −6.0 ± 2.9 nA and −294 ± 149 nA (n = 7), respectively. The traces in red indicate control responses obtained prior to perfusion with STR. Current traces are shown with the 30 s interpulse intervals reduced to 5 s for brevity. **(B)** Methyllycaconitine enhanced responses to ACh in a concentration-dependent manner. Oocytes were stimulated with ACh and the current amplitudes monitored for changes in amplitude during continuous perfusion with MLA at the indicated concentrations. The responses in the presence of MLA (10 nM through 100 μM) were 108 ± 6%, 286 ± 68%, 431 ± 235%, 173 ± 119%, and 90 ± 33% (n = 5), respectively, of control values; responses in the presence of 1 μM were significantly larger than those in the presence of 10 nM MLA. **(C)** Brucine enhanced responses to ACh in a concentration-dependent manner. Oocytes were stimulated with ACh and the current amplitudes monitored for changes in amplitude during continuous perfusion with BRU at the indicated concentrations. The responses in the presence of BRU (100 nM through 300 μM) were 87 ± 16%, 246 ± 74%, 371 ± 101%, 260 ± 102%, and 168 ± 73% (n = 5), respectively, of control values; responses in the presence of 10 μM were significantly larger than those in the presence of 100 nM BRU. **(D)** Acetylcholine responses in oocytes treated with MLA were enhanced by acute perfusion with STR. Oocytes were stimulated with ACh and the current amplitudes monitored for changes in amplitude during continuous perfusion with STR at the indicated concentrations. The responses in the presence of STR (10 nM through 100 μM) were 92 ± 14%, 157 ± 31%, 278 ± 72%, 186 ± 51%, and 51 ± 17% (n = 4), respectively, of control values; responses in the presence of 1 μM were significantly larger than those in the presence of 10 nM STR. **(E)** Acetylcholine responses in oocytes treated with STR were inhibited by acute perfusion of STR. The responses in the presence of STR (10 nM through 100 μM) were 97 ± 2%, 91 ± 8%, 92 ± 12%, 71 ± 18%, and 12 ± 8% (n = 5), respectively, of control values; responses in the presence of 1 μM were no different than those in the presence of 10 nM STR. The estimated IC_50_ for STR was determined to be 20.2 (13.8–29.5) μM. The error bars and the “±” and indicate SD and values in parentheses indicate the 95% CI; “n” indicates the number of oocytes obtained from four donors; ns is not significant. Oocytes in **(B,D)** were incubated with saline containing MLA (20 μM), those in **(C)** with BRU (20 μM), and in **(E)** with STR (20 μM).

During our initial experiments, we observed that currents from oocytes incubated with STR, BRU, or MLA tended to decrease in amplitude over time, but there were differences in the extent to which the currents decayed (Figures 3A–D). Currents from oocytes incubated with MLA decayed to approximately 12% of the initial response to ACh. By contrast, currents from oocytes incubated with STR decayed to 74% of initial amplitudes. Interestingly, currents recorded from oocytes incubated with the STR analog BRU decayed to 15%, a value much smaller than that obtained with STR. To further investigate the enhancing effects of STR on the functionality of α10 nAChRs, we perfused STR-naïve oocytes expressing human α10 nAChRs with STR for 30 min and monitored the current amplitudes in response to ACh. Remarkably, at the end of this perfusion the current amplitudes were enhanced by 518 ± 411% (n = 7) (Figure 4A). Next, we continuously perfused oocytes with STR, BRU, or MLA over a range of concentrations and compared the responses to those recorded prior to ligand perfusion. Oocytes were incubated for 3–4 days in saline containing MLA and consistent with the observations observed during initial experiments, the ACh-evoked currents decayed overtime when perfused with saline alone. Once a stable baseline was achieved, the oocytes were then perfused with increasing concentrations of MLA. Methyllycaconitine increased the amplitudes of ACh-evoked currents at concentrations up to 1 µM but was inhibitory at higher concentrations (Figure 4B). Similar results were obtained with BRU (Figure 4C). Current amplitudes recorded from oocytes pre-incubated with MLA were also enhanced by perfusion with STR (Figure 4D), but by contrast, those from oocytes pre-treated with STR were not enhanced by acute perfusion with STR (Figure 4E).

**FIGURE 5:**
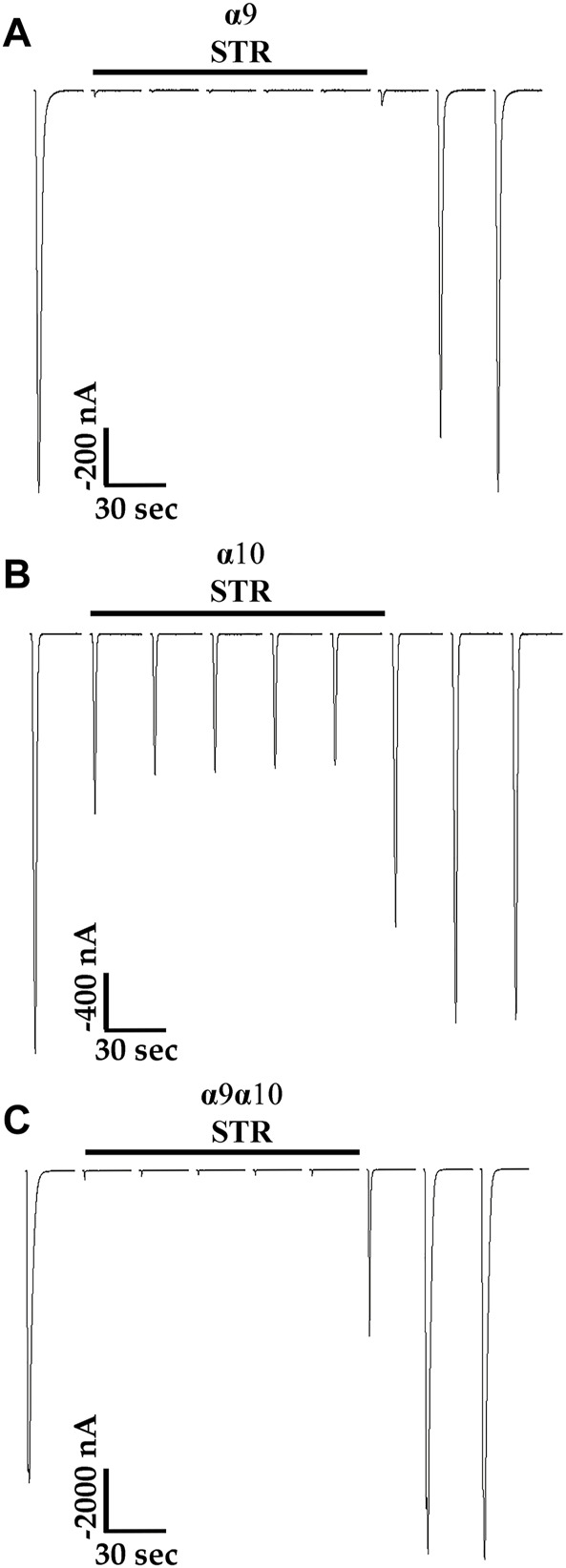
Strychnine is an antagonist of human α9, α10, and α9α10 nAChRs expressed in *X. laevis* oocytes. Oocytes were injected with cRNA encoding either human α9 subunits or α10 subunits to form homomeric subtypes or with cRNA for α9 subunits and α10 subunits together (1:1) to form heteromeric α9α10 nAChRs. Oocytes were then incubated for 3–4 days prior to voltage-clamp electrophysiology. **(A)** Current traces from an oocyte, preincubated with saline containing 5 mM choline, expressing α9 nAChRs. The ACh-evoked currents after acute application of STR (20 μM) were 3 ± 3% (n = 5) of control values. **(B)** Current traces from an oocyte, preincubated with STR (20 μM), expressing α10 nAChRs; the ACh-evoked currents after acute application of STR (20 μM) were 52 ± 13% (n = 5) of control values. **(C)** Current traces from an oocyte, preincubated in saline, injected with cRNA for α9 and α10 nAChRs; the ACh-evoked currents after acute application of STR (20 μM) were 1.0 ± 0.5% (n = 5) of control values. Current traces are shown with the 30 s interpulse intervals reduced to 5 s for brevity, and the horizontal bars above the traces indicate a 5 min perfusion with saline containing STR. The “±” indicates the SD and “n” indicates the number of oocytes obtained from three donors.

Previous reports indicated that STR is an antagonist of mammalian α9-containing nAChRs (Elgoyhen, Johnson et al., 1994; Rothlin, Katz et al., 1999; Baker, Zwart et al., 2004) an effect similar to the results presented in Figure 4E for human α10 nAChRs. Therefore, we also sought to determine the effects of STR on human subtypes closely related to α10 nAChRs including α9 homomers and α9α10 heteromers. Acute STR (20 μM) perfusion of oocytes expressing α9 homomers almost completely inhibited ACh-evoked responses (Figure 5A). By contrast, those from oocytes expressing α10 nAChRs were inhibited by only ∼50% at the same concentration (Figure 5B). Acute perfusion with STR inhibited the ACh-evoked responses in oocytes (incubated in saline) expressing α9 and α10 subunits by ∼99% (Figure 5C).

### Human α9 nAChR function is abolished by STR but enhanced by choline

**FIGURE 6:**
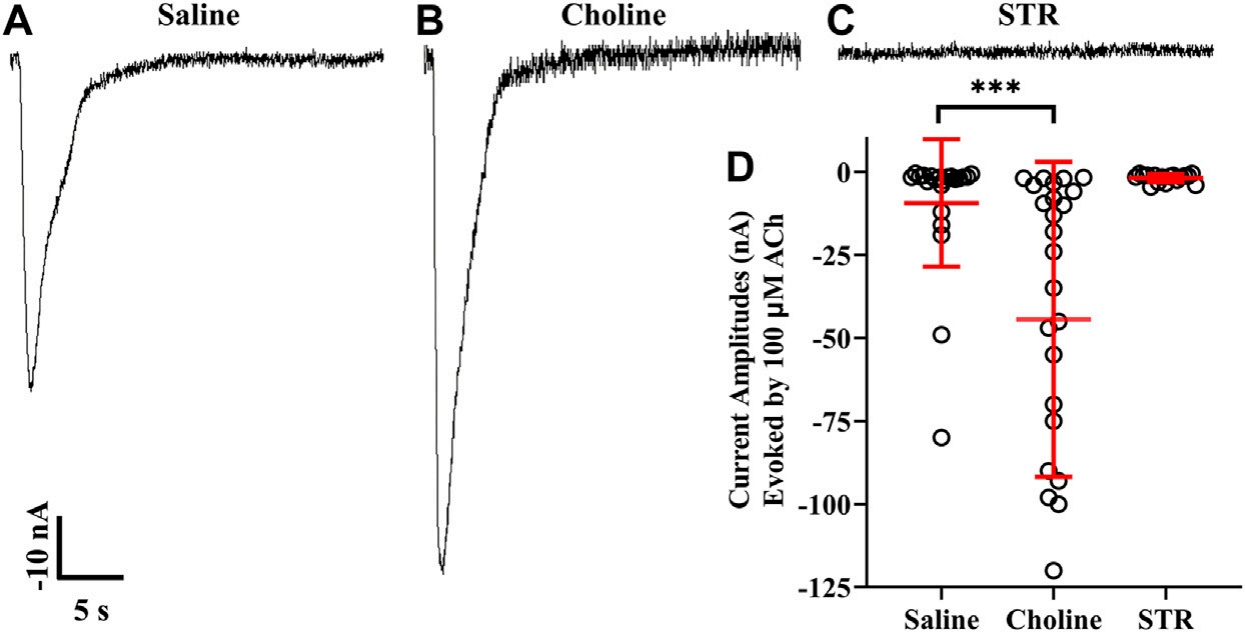
Choline enhances whereas STR inhibits functionality of human α9 nAChRs expressed in *X. laevis* oocytes. Oocytes were injected with cRNA encoding human α9 subunits and incubated for 3 days in frog saline or saline containing choline (5 mM) or STR (20 μM) and assessed by voltage-clamp electrophysiology for functional responses to acetylcholine (ACh; 100 μM). **(A)** A single 30 s current trace from a control oocyte incubated in frog saline. The oocytes responded to ACh with current amplitudes that were −9 ± 19 nA (n = 22). **(B)** Current amplitudes recorded from oocytes incubated with choline were −44 ± 47 nA (n = 25); a subset of this group of oocytes was incubated in frog saline containing STR (20 μM) for 24 h and reassessed for functional responses. The current amplitudes after exposure to STR were reduced to −2.1 ± 1.2 nA compared to −78 ± 44 nA (n = 5). **(C)** Similarly, oocytes incubated with STR for 3 days had ACh responses of −1.8 ± 1.2 nA (n = 17). **(D)** Scatter plot of the data for the experiments shown in **(A–C)**. Current amplitudes from oocytes incubated with choline were significantly larger compared to saline controls. The current amplitude and duration scale-bars apply to all traces. The “±” and the error bars in **(D)** indicate SD. The “n” indicates the number of oocytes obtained from three donors.

The differential results obtained with STR on α9, α10, and α9 plus α10 expressing oocytes prompted us to examine STR effects on homomeric α9 nAChRs. Oocytes injected with cRNA for α9 subunits were incubated in saline or saline containing choline (5 mM) or STR (20 µM). In contrast to the results obtained for α10 nAChRs, choline enhanced functional expression of α9 homomers (Figures 6A,B,D). Strikingly, oocytes incubated in STR did not respond to ACh and no responses were detected even after a 20 min perfusion with saline (Figures 6C,D).

### Snake α-neurotoxins are potent antagonists of homomeric α10 nAChRs

**FIGURE 7:**
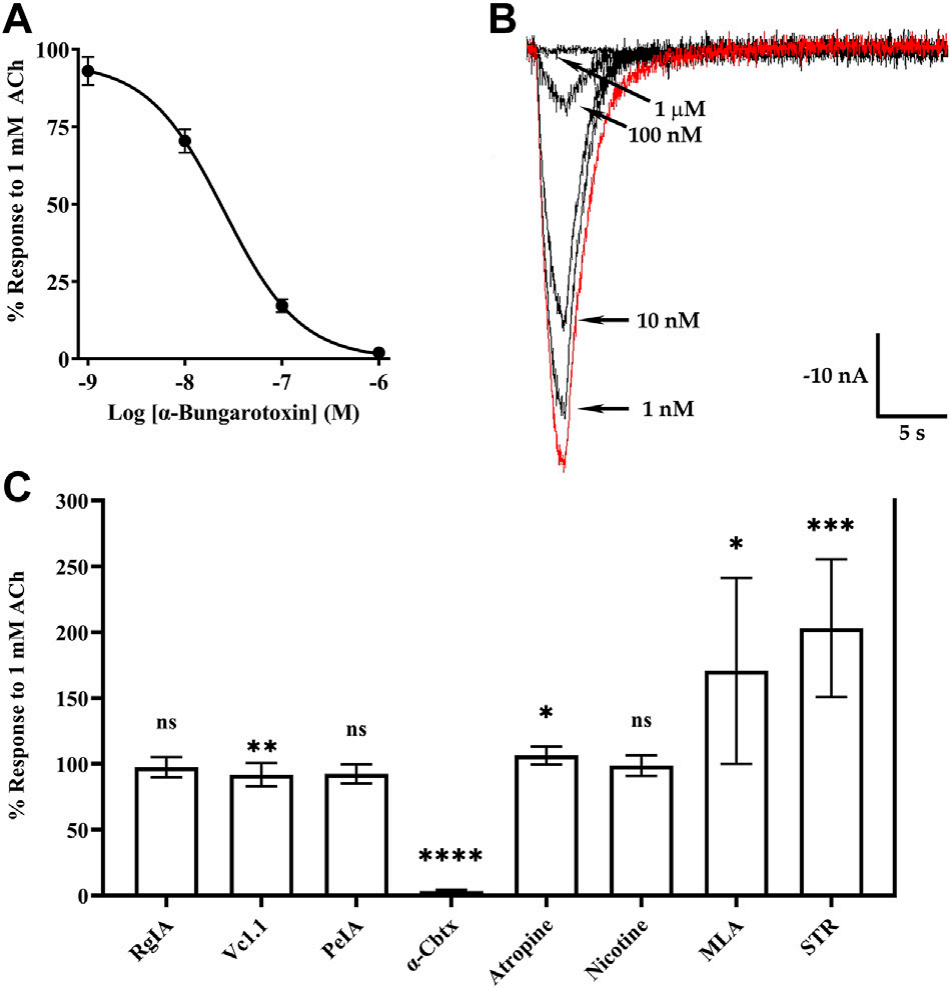
Currents evoked by acetylcholine (ACh; 1 mM) from oocytes expressing human α10 nAChRs are potently inhibited by α-Bgtx and α-Cbtx. *X. laevis* oocytes were injected with cRNA encoding human α10 subunits and incubated for 3–5 days in frog saline containing MLA (20 μM) or STR (20 μM) and assessed by voltage-clamp electrophysiology. **(A)** α-Bungarotoxin inhibited ACh-evoked responses with an IC_50_ of 21 (19–24) nM and the Hill slope was −1.0 (−1.1 to −0.9) (n = 5). **(B)** Current traces showing inhibition of ACh-evoked currents by α-Bgtx; the trace in red indicates a control response to ACh in the absence of α-Bgtx. **(C)** Graph showing the activities of select α-Ctxs, α-Cbtx, atropine, nicotine, MLA, and STR on human α10 nAChRs. The ACh responses in the presence of α-Ctx RgIA were 97 ± 8% (n = 5), 92 ± 9% (n = 9) for α-Ctx Vc1.1, and 97 ± 7% (n = 5) for α-Ctx PeIA. The responses in the presence of α-Cbtx were 3 ± 1% (n = 5). Responses in the presence of atropine, nicotine, MLA, and STR were 106 ± 7% (n = 7), 99 ± 8% (n = 5), 171 ± 71% (n = 8), and 203 ± 52% (n = 8), respectively. All ligands were tested at 10 μM. The parentheses indicate the 95% CI and the error bars in **(A)** and **(C)** indicate SD. The “n” indicates the number of oocytes obtained from nine donors. Oocytes in **(A–B)** were incubated with saline containing MLA (20 μM). In **(C)**, all experiments were conducted on oocytes incubated with STR (20 μM) except those used for testing MLA and STR which were incubated in MLA (20 μM).

**FIGURE 8:**
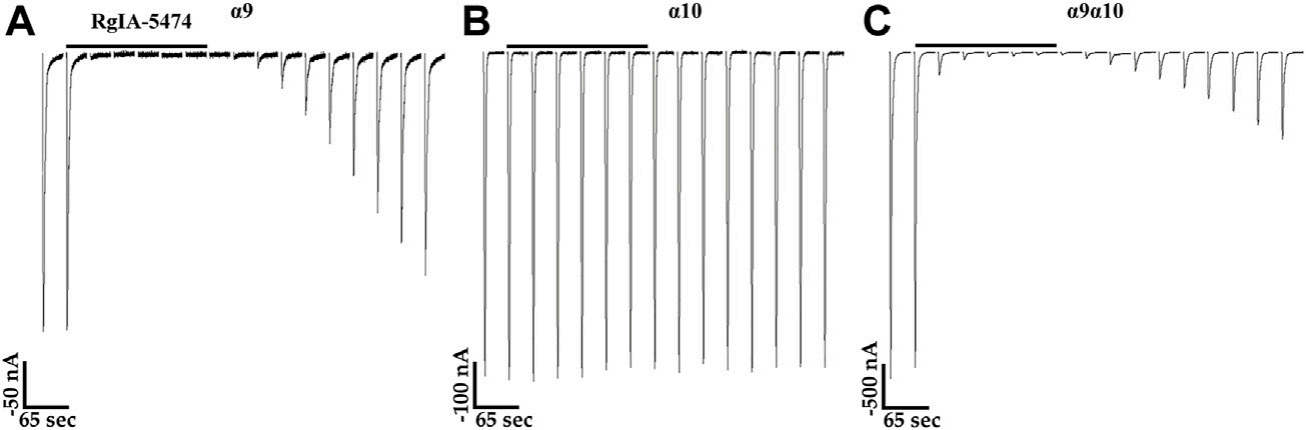
Currents evoked by acetylcholine (ACh) from oocytes expressing human α9 or α9α10 nAChRs are inhibited by α-Ctx RgIA-5474, but those from oocytes expressing α10 nAChRs are not. **(A)** Traces showing inhibition of ACh-evoked (100 μM) currents by RgIA-5474 from an oocyte expressing α9 nAChRs. The responses in the presence of the peptide were 1.0 ± 0.4% (n = 5) of control values. **(B)** Traces from an oocyte expressing α10 nAChRs showing lack of inhibition by RgIA-5474 on ACh-evoked (1 mM) currents. The responses in the presence of the peptide were 100 ± 3% (n = 5) of control values. **(C)** Traces showing inhibition of ACh-evoked (100 μM) currents by RgIA-5474 from an oocyte expressing α9α10 nAChRs. The responses in the presence of the peptide were 3 ± 2% (n = 5) of control values. The horizontal bars above the current traces in **(A–C)** indicate perfusion with saline containing RgIA-5474 (50 nM). Current traces are shown with the 30 s interpulse intervals reduced to 5 s for brevity. The “±” indicates SD and “n” indicates the number of oocytes obtained from three donors. Oocytes in **(A)** were preincubated in saline containing choline (5 mM) and those in **(B)** with STR (20 μM) for 3 days.

The snake toxin α-Bgtx, isolated from the venom of *Bungarus multicinctus,* has been shown to inhibit homomeric human and rat α9 nAChRs and α9α10 heteromers (Elgoyhen, Johnson et al., 1994; Elgoyhen, Vetter et al., 2001; Sgard, Charpantier et al., 2002). Other potent antagonists of mammalian α9**-**containing nAChRs include α-conotoxins (α-Ctxs) PeIA, Vc1.1, and RgIA (Johnson, Martinez et al., 1995; Satkunanathan, Livett et al., 2005; Ellison, Haberlandt et al., 2006). To assess the activity of α-Bgtx on human α10 nAChRs, we obtained a concentration-response curve for inhibition of ACh-evoked responses in oocytes that had been incubated in MLA for 4 days (Figures 7A,B). α-Bungarotoxin inhibited α10 nAChRs with a potency (IC_50_ 21 nM) similar to that previously reported for human α9α10 nAChRs (Sgard, Charpantier et al., 2002). We also assessed a panel of α-Ctxs, atropine, and nicotine that are known antagonists of α9α10 nAChRs as well as a second snake peptide from the cobra *Naja kaokouthia* (Figure 7C). Notably, none of the α-Ctxs, atropine, or nicotine showed substantial antagonist effects on ACh-evoked responses mediated by α10 nAChRs at the concentrations used in this study. However, like α-Bgtx, α-Cbtx nearly eliminated responses to ACh. The alkaloids MLA, STR, and atropine significantly enhanced responses to ACh under these experimental conditions. Lastly, we tested a recently described analog of α-Ctx RgIA that showed pM potency for inhibition of human α9α10 nAChRs (Gajewiak, Christensen et al., 2021). This analog, RgIA-5474, completely inhibited ACh-evoked responses mediated by α9 and α9α10 nAChRs (Figures 8A,C) but had little to no effect on responses from oocytes expressing homomeric α10 nAChRs (Figure 8B).

### Acetylcholine and choline, but not nicotine, are agonists of human α10 nAChRs

**FIGURE 9:**
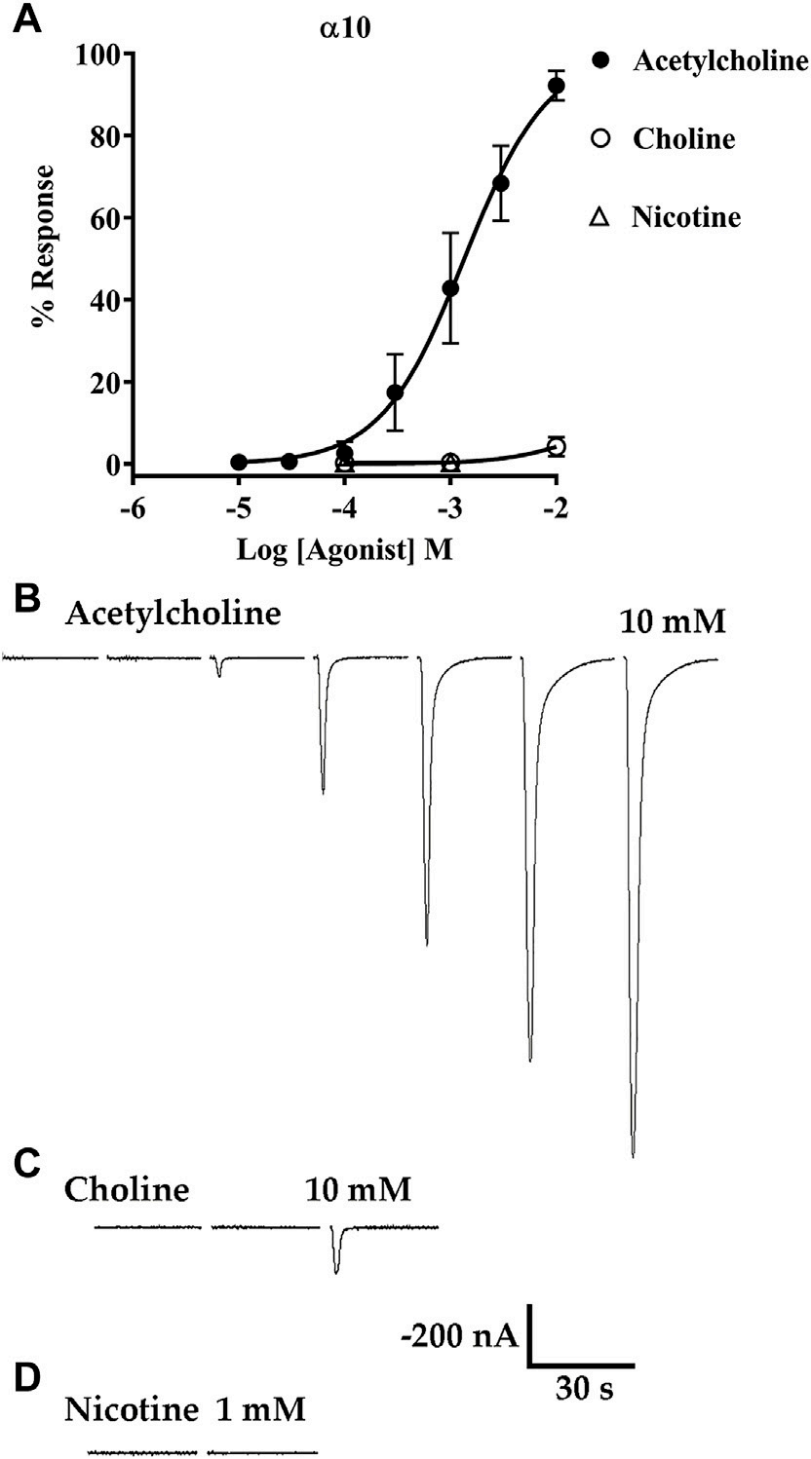
Acetylcholine (ACh) and choline, but not nicotine, evoke currents from oocytes expressing α10 nAChRs. *X. laevis* oocytes were injected with cRNA encoding human α10 subunits and incubated for 3–4 days in frog saline containing STR (20 μM) then assessed by voltage-clamp electrophysiology. **(A)** Concentration-response curve for acetylcholine, choline, and nicotine. The EC_50_ for activation of α10 nAChRs by ACh was 1.35 (1.15–1.58) mM and the Hill Slope was 1.1 (0.9–1.3) (n = 5). Choline was a partial agonist but evoked detectable currents only at 10 mM; the maximal response was 4 ± 2% (n = 5) of the calculated maximal response to ACh. Nicotine failed to induce detectable currents at concentrations up to 1 mM (n = 5). **(B)** Current traces evoked by 10 μM, 30 μM, 100 μM, 300 μM, 1 mM, 3 mM, and 10 mM ACh. **(C)** Current traces evoked by 100 μM, 1 mM, and 10 mM choline. **(D)** Current traces from an oocyte stimulated with 100 μM and 1 mM nicotine. All current traces in **(B–D)** were obtained from the same oocyte and are shown with the 35 s interpulse intervals reduced to 5 s for brevity. The current amplitude and time-scale bars apply to all traces in B-D. Values in parentheses indicate the 95% CI and the “±” indicates SD; “n” indicates the number of oocytes obtained from one donor.

Acetylcholine and choline are agonists of α9 and α9α10 nAChRs, but the canonical nicotinic agonist nicotine has been shown to antagonize α9α10 nAChRs expressed in rat cochlear hair cells and human receptors expressed in *Xenopus* oocytes (Elgoyhen, Vetter et al., 2001; Sgard, Charpantier et al., 2002). To assess the activities of choline and nicotine, first we obtained a concentration-response curve for activation of α10 nAChRs by ACh then the oocytes were stimulated with choline followed by nicotine. Choline evoked small amplitude currents, relative to those evoked by ACh, but only at a concentration of 10 mM (Figures 9A–C). Nicotine however, failed to evoke current responses up to a concentration of 1 mM (Figures 9A–D). These results suggest that physiologically ACh, and not choline, would likely be the primary agonist of homomeric α10 nAChRs.

### Human α10 nAChRs desensitize to ACh faster than α9α10 nAChRs

**FIGURE 10:**
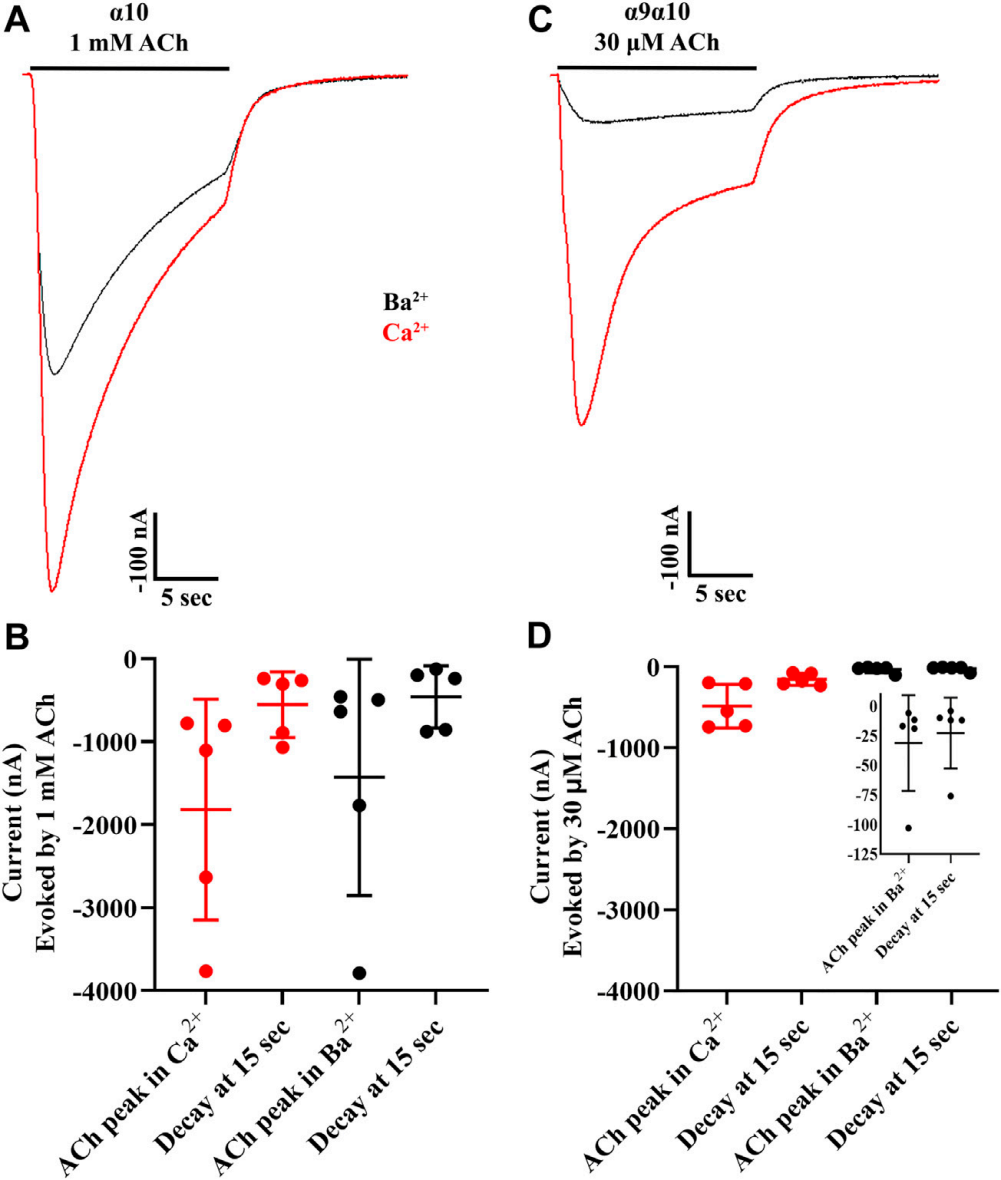
Acetylcholine-evoked currents from human homomeric α10 nAChRs desensitize faster relative to those from human α9α10 nAChRs. *X. laevis* oocytes were injected with cRNA encoding human α10 subunits and incubated in frog saline containing STR (20 μM) or with cRNAs for α9 plus α10 subunits, incubated in saline only, and subjected to voltage-clamp electrophysiology after 3 days. **(A)** Current traces from α10 nAChRs showing the response to a 15 s pulse of ACh in the presence of calcium (red) compared to barium (black). **(B)** The peak current amplitudes in response to ACh were −1,818 ± 1,329 nA (n = 5) in the presence of calcium and −1,430 ± 1,424 nA (n = 5) in the presence of barium. The responses decayed to −553 ± 396 nA or 31 ± 6% (n = 5) of peak amplitude at the end of the 15 s pulse of ACh in the presence of calcium. The responses decayed to −459 ± 375 nA or 35 ± 11% (n = 5) of peak amplitude at the end of the 15 s pulse of ACh in the presence of barium. **(C)** Current traces from α9α10 nAChRs showing the response to a 15 s pulse of ACh in the presence of calcium (red) compared to barium (black). **(D)** The peak current amplitudes in response to ACh were −484 ± 270 nA (n = 5) in the presence of calcium and −31 ± 40 nA (n = 5) in the presence of barium. The responses decayed to −154 ± 74 nA or 33 ± 4% (n = 5) of peak amplitude at the end of the 15 s pulse of ACh in the presence of calcium. The responses decayed to −23 ± 30 nA or 73 ± 8% (n = 5) of peak amplitude at the end of the 15 s pulse of ACh in the presence of barium. The inset shows expanded data for ACh-evoked currents obtained in the presence of barium. The “±” indicates SD and “n” indicates the number of oocytes obtained from two donors.

To examine the desensitization kinetics of α10 nAChRs, oocytes expressing α10 subunits were exposed to 15 s pulses of an EC_50_ concentration of ACh, and the current amplitude at the end of the ACh pulse was compared to the peak current value. For comparison, sibling oocytes expressing α9α10 nAChRs were also examined with 15 s pulses of EC_50_ ACh (Sgard, Charpantier et al., 2002). The ACh-evoked currents were examined in the presence of calcium and also in barium to eliminate the calcium-activated chloride component of the observed response to ACh. Similar to the results presented in Figure 2, peak ACh-evoked currents from α10 nAChRs were reduced in the presence of barium compared to calcium (Figures 10A,B). The responses at the end of the ACh pulse were also smaller compared to peak values as a result of desensitization to the agonist. By contrast with α10 nAChRs, α9α10 currents were much smaller in the presence of barium compared to calcium (Figures 10C,D). Furthermore, α9α10 nAChRs desensitized more slowly than α10 nAChRs.

## Discussion

Under previously tested experimental conditions, mammalian α10 subunits showed no ionic responses to agonists (Elgoyhen, Vetter et al., 2001; Sgard, Charpantier et al., 2002; Fucile, Sucapane et al., 2006; Marcovich, Moglie et al., 2020), and this led to the widely held view that mammalian α10 subunits do not form homomeric receptors and require α9 subunits for function. In this work, we demonstrate that human α10 subunits form homopentameric receptors and display properties distinct from those of the closely related α9 and α9α10 subtypes. For example, the ion channel formed by α10 subunits appeared to show marked differences in permeability to divalent cations compared to α9α10 heteromers (Figure 10). The calcium permeability of α9 homomers and α9α10 heteromers has been measured and shown to be exceptionally high among ligand-gated ion channels (Katz, Verbitsky et al., 2000; Sgard, Charpantier et al., 2002; Fucile, Sucapane et al., 2006). Evolutionary analysis of α9 and α10 subunits revealed that the high calcium permeability of mammalian α9-containing nAChRs is due to three non-synonymous amino-acid substitutions in the α9 subunit (Lipovsek, Fierro et al., 2014). These substitutions are not present in mammalian α10 subunits precluding the formation of a homomeric ion channel with high calcium permeability. Furthermore, human α10 nAChRs are unique in their sensitivity to known ligands of α9 and α9α10 nAChRs. We tested several α-Ctxs, that inhibit human and rodent α9 homomers and α9α10 heteromers including Vc1.1, PeIA, RgIA and its analog RgIA-5474 (Jakubowski, Keays et al., 2004; McIntosh, Plazas et al., 2005; Ellison, Haberlandt et al., 2006; Gajewiak, Christensen et al., 2021), and none of these α-Ctxs showed potent inhibitory effects on α10 nAChRs (Figures 7, 8). We also tested the muscarinic receptor antagonist atropine as well as nicotine and found a similar lack of inhibitory activity (Figure 7). In fact, α10 currents from oocytes exposed to atropine were significantly larger than baseline controls suggesting that this alkaloid may also enhance function of α10 nAChRs. Evolutionary analysis of chick and mammalian α10 subunits concluded that the low potency and partial efficacy of choline for activation of mammalian α9α10 nAChRs can be attributed to non-conserved amino-acid residues in the α10 subunit (Moglie, Marcovich et al., 2021). The low efficacy of choline for activation of human α10 nAChRs is consistent with these observations (Figure 9). By contrast, choline is a full agonist of chick α10 nAChRs again due to non-conserved amino-acid residues in the ACh-binding pocket (Moglie, Marcovich et al., 2021).

There are substantial similarities in the amino-acid sequences of mammalian α10 and α9 subunits, and the latter have previously been shown to form functional homomers (Elgoyhen, Johnson et al., 1994; Sgard, Charpantier et al., 2002; Fucile, Sucapane et al., 2006; Filchakova and McIntosh 2013). Mammalian α10 subunits are also quite similar in sequence to chick α10 which also assemble as functional homomers (Marcovich, Moglie et al., 2020). Despite these similarities, functional mammalian α10 homopentamers have not been previously reported. Several lines of evidence suggest that there are features of mammalian α10 and α9 subunits that impair function when expressed as homomers. Firstly, human α9α10 heteromers express well in oocytes, but, by contrast, α9 homomers express poorly or not at all (Filchakova and McIntosh 2013). The fact that when the two subunits are expressed together results in increased function compared to α9 or α10 alone suggests that α9α10 heteromers might be assembled more efficiently. However, binding studies using ^125^I-α-Bgtx demonstrated that human α9 homomers are assembled and inserted in the oocyte membrane at levels similar to α9α10 heteromers, but very few of them show canonical ionic function (Sgard, Charpantier et al., 2002). Alternatively, human α9 and α10 subunits when expressed together might result in a larger fraction of the receptors expressed in a conformation that can be gated by agonists for ionic functions. Secondly, chimeric constructs containing the extracellular ligand-binding domain of the mammalian α10 subunit and the transmembrane and cytoplasmic domains of the serotonin 5-HT_3_ receptor have been shown to express as homomers in oocytes and human embryonic kidney cells (Baker, Zwart et al., 2004) suggesting that some element of the transmembrane or cytoplasmic domains may impair function. Such impairments have been shown for human nAChRs containing α6 subunits which express poorly, or not at all, in oocytes and cell lines (Kuryatov, Olale et al., 2000; Evans, Bose et al., 2003). Poor expression of α6-containing nAChRs was overcome by replacing the transmembrane and cytoplasmic regions with those of the closely related α3 subunit (Kuryatov, Olale et al., 2000; McIntosh, Azam et al., 2004). Enhanced expression of α6-containing nAChRs was also found when the first cytoplasmic loop of α6 was replaced with that of α3 (Jensen, Hoestgaard-Jensen et al., 2013; Ley, Kuryatov et al., 2014). Collectively, expression studies of α6, α9, and α10 indicate that structural elements of some nAChR subunits can potentially inhibit expression of receptors in a state that can be gated by agonists for ionic function.

Receptor function may also be dependent on or be regulated by other cellular proteins as demonstrated for the lynx1 prototoxin which modulates the expression and function of several nAChR subtypes (Ibanez-Tallon, Miwa et al., 2002; George, Bloy et al., 2017; Parker, O'Neill et al., 2017; Miwa 2021). Recently it was shown that certain transmembrane proteins found in native cells associate with α6 and α9 subunits and enable heterologous expression of functional nAChRs (Gu, Matta et al., 2019; Gu, Knowland et al., 2020). In the case of α9-containing nAChRs, transmembrane protein of the inner ear (TMIE) was found to synergize with choline acetyltransferase to enable functional expression of human α9 homomers and α9α10 heteromers in human embryonic kidney cells. However, although choline acetyltransferase alone facilitated surface expression of α9α10 heteromers, ionic function required TMIE. Interestingly, several members of an orphan family of transmembrane proteins (TMEM) unrelated to TMIE were shown to facilitate ionic functions of human α9α10 heteromers and appeared to show α10-dependent effects. Speculatively, α10 homomers may also require an endogenous protein for ionic functions. Lastly, Gu et al., also reported that ionic responses in cells transfected with α9α10 plus TMIE were enhanced by incubation with ACh, MLA, or α-Bgtx suggesting that external ligand-binding can also enhance function. Thus, an endogenous protein or ligand might be used by certain cells to respond to environmental conditions in order to modulate the functional properties of α10 nAChRs according to cellular needs.

Here we show that ionic responses mediated by human α10 nAChRs were obtained by incubation with the membrane impermeant ligand α-Bgtx, as well as STR, BRU, or MLA (Figure 1). Although the conditions used in this study are not physiological, they may have pathophysiological relevance. Strychnine and BRU are regularly ingested as non-traditional medicine in the form of kuchula seeds, and concentrations of BRU and STR in the nM range showed activity on α10 nAChRs (Figures 4C,D). Strychnine also affected the activity of α9 homomers and α9α10 heteromers (Figures 5, 6). Therefore, multiple systems that express α9 and α10 subunits could be affected by ingestion of kuchula seeds. We also show that human α9 nAChR responses, but not those of α10, could be enhanced by incubation with choline (Figures 1, 6) which may correlate with ligand activity; choline is an agonist of α9 homomers with µM potency (Sgard, Charpantier et al., 2002) but only partially activates α10 nAChRs at 10 mM (Figure 9). This discovery may facilitate studies that examine the pharmacological properties of homomeric human α9 nAChRs, studies that have historically been difficult to conduct. Exposure to STR produced differential effects depending on the nAChR subtype and ligand-incubation procedures. Ionic function of α10 nAChRs was enabled by acute STR exposure which produced progressively larger currents over the course of 30 min in oocytes that had been previously incubated in saline only (Figure 4A). By contrast, acute STR perfusion of oocytes expressing α10 nAChRs and pre-treated with STR resulted in inhibition of ACh-evoked currents (Figure 5). Strychnine inhibition was also observed for α9 homomers and α9α10 heteromers. These results may indicate that STR interacts with at an external site on the receptor. Alternatively, STR and other membrane permeant alkaloids might cross the cell membrane and modulate ionic functions or promote receptor insertion into the plasma membrane. An allosteric ligand-binding site has been proposed for the α9 subunit (Zorrilla de San Martin, Ballestero et al., 2007) and the closely related α7 nAChR (Bertrand, Bertrand et al., 2008; daCosta, Free et al., 2011). Future studies are needed to determine if the effects of alkaloid ligands on α10 nAChR function are consistent with interaction with a positive allosteric modulatory site. It is noteworthy that ligands that bind to the allosteric site of α7 nAChRs produce differential effects on receptor function. For example, the allosteric modulator NS6740 is capable of inducing two different conformational states of α7 nAChRs, one of which is a prolonged non-activatable state (Papke, Bagdas et al., 2015). Similarly, STR abolished function of human α9 nAChRs, an effect that lasted for the duration of the experiment (Figure 6) in stark contrast to results obtained for α10 nAChRs. In this way, different nAChR subtypes might be selectively recruited from mixed populations via ligand binding. Indeed, STR incubation of oocytes co-expressing α9 and α10 subunits produced a population of receptors that was resistant to inhibition by acute exposure to STR or RgIA-5474 (data not shown). These results are reminiscent of studies of the lynx1 protein that was shown to change the functional properties and expression patterns of human α3β4-containing nAChRs (George, Bloy et al., 2017).

The quiescent nature of human α10 nAChRs expressed in oocytes may occur in native cells. To our knowledge, ionic currents mediated by native α10-containing nAChRs have thus far only been recorded in hair cells of rodent inner ear (Elgoyhen, Johnson et al., 1994; McIntosh, Plazas et al., 2005; Ellison, Haberlandt et al., 2006; Lipovsek, Marcovich et al., 2021). However, α10 subunits have been implicated in non-ionic nAChR responses in immune cells (Peng, Ferris et al., 2004; Mikulski, Hartmann et al., 2010; Mishra, Rir-sima-ah et al., 2010; Richter, Mathes et al., 2016). These immune cells studies provide evidence that some nAChRs function in a metabotropic-like state rather than one with ionic functions. Immune cell nAChRs include those assembled from α7, α9, or α10 subunits and form part of a cholinergic anti-inflammatory system (Tracey 2007; Fujii, Mashimo et al., 2017; Alen 2022). The present study suggests the possibility that some nAChRs might be expressed in a quiescent state, but canonical ion channel functions can be induced by certain ligands. Indeed, there is precedent for native cell expression of quiescent nAChRs that can be pharmacologically converted to an ionic conformation by allosteric modulators. Rat adrenal chromaffin cells abundantly express α7 nAChRs, but a large fraction of the receptor population is evidently present in a non-activatable state (Hone, Rueda-Ruzafa et al., 2020); see also (Higgins and Berg 1988). In fact, in some chromaffin cells α7-mediated responses could only be observed in the presence of the allosteric modulator PNU-120596. Similar results have been found for bovine and human adrenal chromaffin cells (del Barrio, Egea et al., 2011; Perez-Alvarez, Hernandez-Vivanco et al., 2012).

In human epidermis, expression of α10 and α9 subunits often do not parallel each other, and therefore it is possible that homomeric receptors are present in these tissues (Kurzen, Wessler et al., 2007). In dorsal root ganglion neurons molecular biology assays consistently detect mRNA and protein for α10 but, in some cases, not for α9 subunits though ionic currents attributable to α10-containing nAChRs have not been identified (Haberberger, Bernardini et al., 2004; Rau, Johnson et al., 2005; Bschleipfer, Nandigama et al., 2012; Hone, Meyer et al., 2012; Zhang, Hartung et al., 2019). Thus, it remains unknown whether α10 nAChRs expressed in the absence of α9 subunits would function in an ionic or in a metabotropic-like state. The discovery that ligand-binding can enable the ionic functions of α10 nAChRs should greatly facilitate the development of α10 homomer-specific ligands. Such ligands can then be used to resolve questions concerning the native expression of α10-containing nAChRs and their roles in human physiology and pathology.

## Data Availability

The raw data supporting the conclusions of this article will be made available by the authors, without undue reservation.
